# Evaluation of the Peri-Implant Tissues of Patients with Severe Bone Atrophy Treated with a New Short and Extra-Short Implant System—A Pilot Study

**DOI:** 10.3390/jfb15100288

**Published:** 2024-09-29

**Authors:** Kely Cristina de Moraes, Geninho Thomé, Flávia Noemy Gasparini Kiatake Fontão, Carolina Accorsi Cartelli, Rosemary Adriana Chierici Marcantonio, Carolina Mendonça de Almeida Malzoni, Elcio Marcantonio Junior

**Affiliations:** 1 Odontology at Ilapeo College, Curitiba 80710-150, Brazil; kelycmoraes@hotmail.com (K.C.d.M.); geninho@ilapeo.com.br (G.T.); fgaspar@ilapeo.com.br (F.N.G.K.F.); carolina.accorsi@hotmail.com (C.A.C.); 2 Department of Diagnosis and Surgery, School of Dentistry of Araraquara, São Paulo State University (Unesp), Araraquara 14801-385, Brazil; adriana.marcantonio@unesp.br (R.A.C.M.); ca.malzoni@gmail.com (C.M.d.A.M.)

**Keywords:** short implants, extra-short implants, bone loss, implant-supported prosthesis, soft tissue

## Abstract

This study aimed to assess clinical and radiographic outcomes, including implant survival, marginal bone loss, and patient satisfaction, in individuals with severe bone atrophy treated using a newly developed system of short and extra-short implants. A total of 44 implants (37 short and 7 extra-short) were placed with immediate loading in 11 patients. The patients were followed up at between 6 and 24 months. Bone changes, keratinized mucosa, bleeding on probing, probing depth, crown-to-implant ratio, and patient satisfaction were evaluated. An implant survival and success rate of 100% was observed. The peri-implant bone condition showed no significant associations between marginal bone loss (MBL) and gingival recession. In extra-short implants, the crown-to-implant ratio did not affect MBL in the evaluated times. However, short implants showed a statistically significant inverse correlation between mesial measurement and crown-to-implant ratio (*p* = 0.006) and between distal measurement and crown-to-implant ratio (*p* = 0.004) over six months. Plaque was present in the mesiobuccal regions in 38.64% of the implants, with extra-short implants having the highest relative frequency (71.4%). Bleeding was observed in 18.9% of the short implants in the mesiolingual region and 14.3% of the extra-short implants. There was a statistically significant association between bleeding on probing in the mesiobuccal region and the type of implant (*p* = 0.026). The analysis of probing depth showed no difference between the types of implants. Within the limits of this study, short and extra-short implants presented similar clinical and radiographic behavior of soft and hard tissues in the evaluated times.

## 1. Introduction

Modern dental implantology requires solutions that reduce morbidity and treatment time. At the same time, challenging situations, such as vertical bone atrophy, demand advanced technology and designs, surgical efficiency, and intelligent prosthetic solutions.

Maxillary atrophy is a frequently encountered condition that makes implant-supported oral rehabilitation difficult. This deficiency is directly related to tooth loss time and/or severity [1]. It can lead to the proximity of the bone defect to critical anatomical structures, making it impossible to place implants with conventional lengths without prior bone grafting [2].

The anatomical limitations that make it difficult to place implants in the maxillary bone include the maxillary sinus pneumatization [3], nasal fossa, inferior alveolar nerve, mental nerve, and mandibular fovea. Bone atrophy and proximity to these regions require prior surgical procedures to allow the placement of regular-size implants such as bone grafts, distraction osteogenesis, and inferior alveolar nerve transposition [1,4,5,6]. However, these treatments offer increased morbidity, edema, treatment time, cost, and risk of paresthesia [1,7,8,9,10,11]. Short and extra-short implants have been developed to address these disadvantages as a viable treatment alternative [12,13].

Oral rehabilitation with short and extra-short implants in atrophic areas is a treatment option with less complexity, trauma, cost, and treatment time [1,5]. However, attention must be paid to the technique used to place these implants, as they offer a smaller bone-to-implant contact area. Therefore, factors such as bone density, quality and quantity, implant surface characteristics, surgical technique, prosthesis mechanical conditions, tissue biotype, and occlusal balance must be meticulously planned and adjusted during the treatment [5,14,15].

Studies evaluating prosthetic aspects associated with implant size are still limited. Misch et al. (2006) [16] concluded that the length of the implant does not seem to be the most critical factor in the distribution of loads at the bone–implant interface. For these authors, the diameter of the implant, increase in the crown-to-implant ratio (C/I), increase in the chewing forces, and lateral tensions are important factors for the distribution of loads and could lead to possible implant failures. Anitua et al. (2014) [4], Anitua et al. (2015) [17], and Di Fiore et al. (2019) [18] also indicated that implants without a positive crown-to-implant ratio are more susceptible to failure as they influence the increase in marginal bone loss (MBL).

However, since the short implants are two-piece, the occlusal adjustment could affect the intensity of stress transmitted to the marginal bone and proximity to the microgap, in addition to repeated abutment tightening/loosening [19]. These associated or isolated factors can influence marginal bone loss and promote the inflammation and accumulation of microorganisms in short implants, including occlusal maladjustment [12].

In the literature, some authors [5,10,20] compared regular-size implants with short implants; however, few observed the behavior of extra-short implants in regions of extreme atrophy. Thus, there is much to be explored regarding the mechanical and biological mechanism of implants with reduced sizes [5].

Recently, the Neodent company launched a new model of short and extra-short implants with important changes to their design. Different classifications [6,21] were proposed for short (≤6 mm) and extra-short (≤5 mm) implants [22]. In this study, we consider short (≥8.5 mm ≤6 mm) and extra-short (<6 mm) implants.

In this way, this study aimed to assess clinical and radiographic outcomes, including implant survival, marginal bone loss, and patient satisfaction, in individuals with severe bone atrophy treated using a newly developed system of short and extra-short implants.

## 2. Materials and Methods

### 2.1. Study Design

This clinical study was previously approved by the Research Ethics Committee of the Universidade Tuiutí do Paraná (Curitiba, Brazil no. 5.793.029).

The inclusion criteria were patients who had received Short^®^ system implants (Neodent, Curitiba, Brazil) with prostheses installed at least six months before the data collection at Faculdade ILAPEO (Curitiba, Brazil), patients with periapical radiography performed at the time of prosthesis installation, and patients with good systemic health.

Patients with no periapical radiography at the time of prosthesis installation, a lack of follow-up visits, the presence of systemic diseases that could affect tissue response, uncontrolled diabetes, the use of bisphosphonates or proton inhibitors (PPI), a history of radiotherapy treatment, and autoimmune diseases were excluded.

After analyzing the medical records, participants were selected and invited to participate. All patients signed the informed consent form before any study procedure. Twelve patients were selected, one of whom did not attend appointments and was therefore excluded. Thus, 11 adult patients of both sexes with partial or total maxillary edentulism were included. These patients received 44 implants, 37 short implants (≤8.5 mm and ≥6 mm), and 7 extra-short implants (<6 mm) and were rehabilitated with total or partial implant-supported prostheses (temporary and/or metal-ceramic). The patient follow-up was carried out at a minimum of six months and a maximum of 24 months after the procedure. All patients were on a maintenance program with appointments every six months.

### 2.2. Surgical Procedures

Short^®^ implant (Neodent, Curitiba, Brazil) was placed under local anesthesia (4% Articaine with 1:100,000 epinephrine) and with adequate bone bed preparation according to the manufacturer’s recommendations. All patients received the same brand and implant model with variations in the implant length and diameter according to the patient’s needs. Only one operator was responsible for all treatments. The patients were also given post-operatory and oral hygiene orientations. Patients with parafunction were also oriented to search for treatment and use an occlusal splint. However, the study team did not deliver the treatment.

After implant placement, the suture was performed, and an X-ray was taken. Patients were instructed to return between 7 and 14 days after surgery to remove the sutures.

The loading protocol (delayed or immediate) was selected according to each patient’s needs and the manufacturer’s instructions (IFU). At the surgeon’s discretion, immediate loading was applied when primary stability reached at least 35 N.cm and the patient presented physiological occlusion.

All patients received a temporary or final prosthesis per the clinician’s definition. After the prosthesis installation, a radiographic examination was performed to confirm the adaptation of the prosthetic work.

### 2.3. Clinical Analysis

The following variables (a and b) were extracted from the patient’s medical records and (c) clinically collected during the follow-up visit:

(a) Variables related to the patient: sex, age, systemic conditions, smoking, signs of parafunction, history of diseases, regularity of follow-up visits, and follow-up time.
(b) Variables related to the implant treatment: type and size of implants, need for grafts, bone type, type of implant load, and abutments.
(c) Outcome variables: mechanical complications (wear and fractures of prostheses; abutments and implants; and screw loosening, replacement, and loss), biological complications (presence of biofilm, bleeding on probing, suppuration, gingival recession, hyperplasia, bone loss), implant condition (pain, mobility), crown-to-implant ratio, and patient satisfaction.

The data described in item C were obtained according to the following steps: removal of the restorative material from the screw holes; assessment of prosthesis screw tightness; prosthesis removal; evaluation of the presence of plaque; assessment of abutment tightness; examination of the condition of the implants and peri-implant tissues; periapical radiography performed to check the peri-implant bone condition (presence or not of bone loss); and a questionnaire about satisfaction with the treatment performed.

After collecting clinical data, patients received prophylaxis, instructions in effective oral hygiene, guidance on a regular maintenance protocol every six months, and, if necessary, the prostheses were reinstalled with occlusal adjustments.

### 2.4. Peri-Implant Soft Tissue Analysis

The presence of visible plaque (VP) in the abutments and prosthesis was evaluated with Score 0 indicating absence of plaque and Score 1 indicating its presence. Bleeding on probing (BP) [23] and probing depth (PD) were assessed at six different sites: mesio-buccal, buccal, disto-buccal, mesio-lingual, lingual, and disto-lingual.

The height of the keratinized mucosa (KM) was evaluated measuring the distance from the gingival margin to the mucogingival line in all implants in the mesiobuccal, buccal, and distobuccal positions. The measurements were classified as 0, >0 <2, and ≥2 mm. The gingival phenotype was classified as thick or thin through the visual analysis of the millimeter probe transparency in the peri-implant sulcus.

The appearance of the peri-implant mucosa was visually evaluated concerning the presence or absence of hyperplasia, with Score 0 being assigned for its absence and Score 1 for its presence. Gingival recession was also assessed visually by identifying the exposure of the abutment transmucosal or implant threads, then assigning Score 0 for the absence of recession and Score 1 for the presence of recession. Suppuration was assessed by applying digital pressure to the peri-implant tissues, with Score 0 indicating its absence and Score 1 indicating its presence.

All analyses were performed by a single experienced operator calibrated in all measurements.

### 2.5. Analysis of the Implant and Prostheses Survival and Success Rates

To evaluate the implant success, the absence of peri-implant infection, continuous radiolucency around the implant, bone loss in the first year < 1.5 mm, and annual bone loss < 0.2 mm were radiographically analyzed [24]. Furthermore, pain, mobility, and percussion tests were also performed using a clinical mirror handle and prosthetic installation screwdriver, according to the criteria of Albrektsson et al. (1986) [25]. The bone type was collected from clinical follow-up records according to the classification of Lekholm and Zarb (1985) [26].

The prosthesis was considered successful when it was functional without presenting more than four complications that could be resolved. Complications included prosthesis, implant, and abutment wear, chips, and fractures and screw loosening, replacement, and loss. The loss of the abutment and abutment screw torque was evaluated with a counterclockwise force of 10N.

The crown-to-implant ratio (C/I) was split into two groups (C/I < 2 and C/I ≥ 2) and was analyzed and correlated with marginal bone loss (MBL), prosthesis design and material over time. The clinical C/I was calculated according to below [4,17,18]:

(crown length + MBL) ÷ (implant length − MBL) (1)

### 2.6. Radiographic Assessment

**Figure 1:**
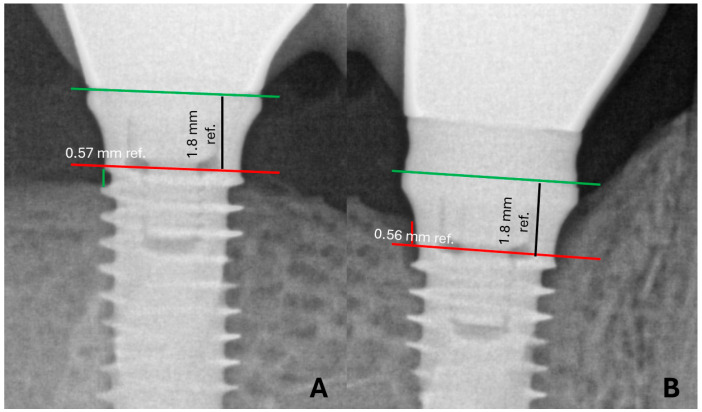
Methods for measuring bone height in an intraoral X-ray image of a lower implant. (**A**) Measurement below the implant platform and (**B**) above.

Intraoral radiographs were taken at the time of prosthesis installation (T0) and follow-up visit (T1) using the Heliodent X-ray device (Sirona, Bensheim, Germany), with a CMOS sensor (Xios Supreme, Sirona, Bensheim, Germany). The periapical parallelism technique was used with an XCP-DS positioner (Dentsply Rinn, Elgin, IL, USA) to obtain radiography with standardized distance. After image calibration using the implant diameter as a reference, linear mesial and distal peri-implant bone height measurements were performed using the Sidexis 4 Software (Sirona). A reference line was drawn on the implant platform in the calibrated image. The measurement was obtained from the most apical point of the radiolucent image (at the bone/implant interface) to the implant platform reference line for implants with bone level below the implant platform line (Figure 1A). In implants with bone level above the implant platform, the measurement was performed from the highest point of the alveolar crest to the implant platform line (Figure 1B).

### 2.7. Assessment of Patient Satisfaction

Patient satisfaction with the treatment performed was assessed through a questionnaire applied as a visual analogue scale (VAS), with gradations from 0 to 10, with 0 being “totally dissatisfied” and 10 being “totally satisfied” [27].

### 2.8. Statistical Analysis

Descriptive data analysis was carried out with estimates of the mean, median, standard deviation, and 25% and 75% percentile of quantitative variables and simple and relative frequencies of qualitative variables.

All analyses were conducted in the R 4.1.0 environment [28].

## 3. Results

The retrospective sample consisted of 11 patients (8 female and 3 male) with an average age of 65 years (varying from 46 years to 79 years). A total of implants were placed, 37 short (7 mm, 7.5 mm, and 8 mm in length) and 7 extra-short (5.5 mm in length), with an average implant follow-up time of 13.45 months. Of these 11 patients, 72.73% had systemic alteration (controlled), 81.82% were using medication, and only 27.27% were smokers. All patients regularly went to follow-up visits. The average number of implants placed per patient was four. Eight implants were placed in bone type I, twenty in type II, and sixteen in type III. The implants were loaded immediately (9; 81.82%) and early (2; 18.18%) (Table 1).

No implants presented pain or mobility, resulting in an implant success rate of 100%. An implant success rate of 100% was observed. The patients’ maxillo-mandibular conditions were varied. Nine patients (90.91%) had total edentulism. They were treated with fixed implant-supported rehabilitation and hybrid prostheses in the upper and lower regions. Two (9.09%) patients had partial edentulism, both of whom received single screw-retained dentures with two elements in the same lower dental arch; 90% of the patients did not change their prosthesis teeth; and 63.64% received a hybrid prosthesis (Table 2).

The prosthesis survival and success rate was 100%.

### 3.1. Prosthesis and Mechanical Complication Analysis

The most found mechanical complications were loss of prosthetic screw torque (n = 2; 4.55%), loss of abutment screw torque (n = 2; 4.55%), and dental wear (n = 2; 4.55%) during the observational period. In these cases, the complications were caused by dental clenching and occlusal maladjustment, which led to the loosening of screws and abutments. Both loosening was identified early (6 months) after prostheses installation. Occlusal adjustments were performed at the end of the follow-up visits on two prostheses. There was no prosthetic or abutment screw fracture. No metallic, ceramic, or acrylic prostheses substructure fractures were observed.

### 3.2. Crown-To-Implant Ratio (C/I)

Overall, the average crown height was 13.33 mm. Most of the mesial and distal C/I was higher than 2 (59.09% and 69.65%, respectively). The mean mesial C/I was 2.11 ± 0.72, and the mean distal C/I was 2.16 ± 0.57 (Table 3).

### 3.3. Marginal Bone Loss—MBL

Marginal bone loss was higher in short implants with a mesial bone loss of 0.73 mm, and distal of 0.86 mm. Regarding the extra-short implants, the mesial and distal bone loss values were −0.1 and 0.2, respectively (Table 4).

#### Peri-implant soft tissue conditions

**Figure 2:**
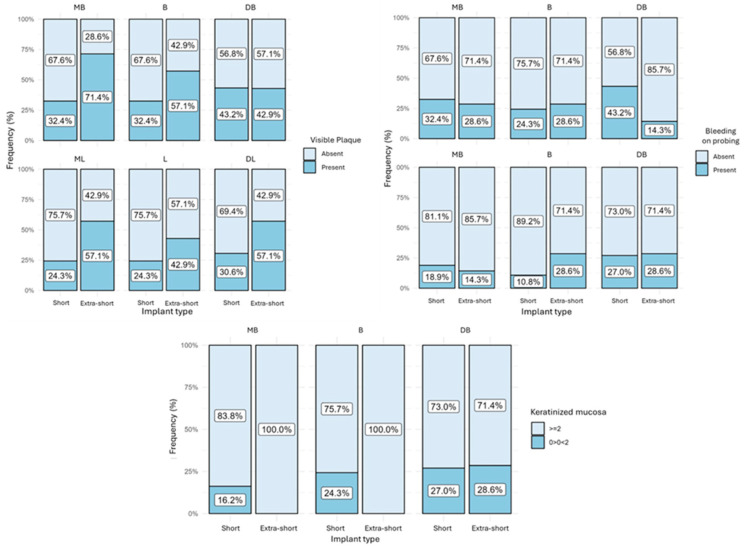
Bar graph of the percentage of visible plaque, bleeding on probing, and keratinized mucosa presence according to implant type in each region.

**Figure 3:**
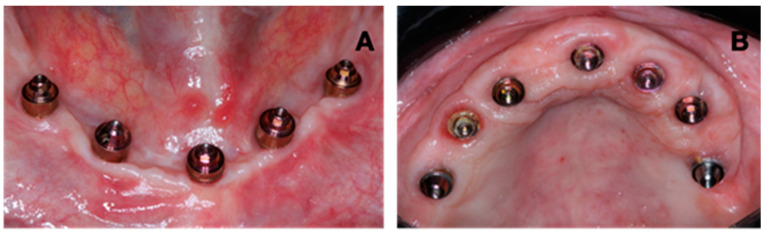
Visible plaque (VP) in intermediates. (**A**) Patient with absence of VP (Score 0). (**B**) Patient with presence of VP (Score 1).

**Figure 4:**
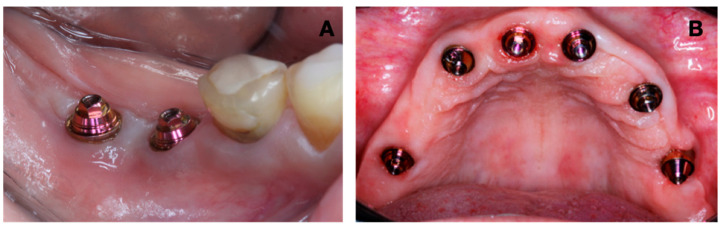
Bleeding on probing (BP). (**A**) Patient with absence of BP (Score 0). (**B**) Patient with presence of BP (Score 1).

Visible plaque was observed in all regions analyzed. The extra-short implants in the MB region had the highest quantity of implants with plaque (71.4%), and the lowest was in the ML and L regions of short implants (24.3%) (Figure 2 and Figure 3). Regarding bleeding on probing, short implants presented BP in 43.2% of the implants in the DB region (Figure 2 and Figure 4). Most of the short and extra-short implants presented keratinized mucosa ≥ 2 mm (Figure 2).

**Figure 5:**
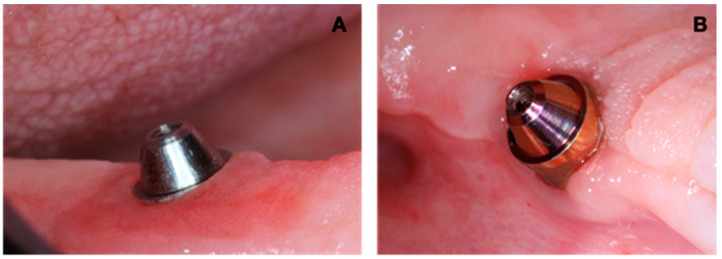
Gingival recession. (**A**) Patient with its absence (Score 0). (**B**) Patient with its presence (Score 1).

The short implant group had an equilibrium between thin and thick gingival phenotype; in the extra-short, most had thick phenotype (Table 5). Gingival recession was present in eleven (29.8%) short implants and one (14.3%) extra-short implants (Figure 5).

The highest mean probing depth was found in the mesiobuccal region (2.43 mm) and the lowest was observed in the disto-lingual region (1.76 mm) in short implants. In the extra-short implants, the highest mean probing depth was also found in the mesiobuccal region (2.71 mm) and the lowest was observed in the lingual region (1.14 mm) (Table 6).

### 3.4. Patient Satisfaction

**Figure 6:**
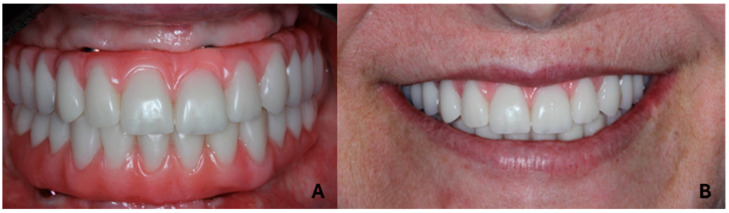
Final rehabilitation results. (**A**) Intraoral view. (**B**) Extraoral view.

All patients (100%) were completely satisfied with the proposed treatment regarding chewing quality and capacity and the prostheses smile. Regarding satisfaction with prosthesis cleaning and their prosthesis when speaking, the results showed 36.36% low satisfaction and 81.82% good satisfaction. Final rehabilitation is shown in Figure 6.

## 4. Discussion

With the evolution of implant dentistry, modifications in the implant design were proposed to meet the specificities of the treatment and allow oral rehabilitation with lower cost, morbidity, and surgical time [4,17,29,30]. However, the ideal definition of regular, short, and extra-short implants is still inconclusive in the literature. Previous studies [10,31] considered regular implants to be ≤11 mm, short implants to be <7 mm, and extra-short implants to be ≤6.5 mm. In this study, short implants were considered to be 8.0 mm and extra-short implants were considered to be 4.0 and 5.5 mm in length.

Several authors have concluded that reduced-size implants offer an effective solution for patients with severe maxillary atrophy, achieving high long-term survival rates between 92% and 99% [1,15,32,33,34]. These results corroborate those of the present clinical study, where the implant survival was 100%. Randomized clinical trials (RCTs) evaluating and comparing the behavior of short implants in atrophic areas and long implants in grafted areas in the posterior region of the mandible for one year [29], three years [30], and five years [20] of follow-up showed similar results between both treatments, suggesting that treatment with short implants is a feasible alternative to bone graft associated with conventional implants and is associated with shorter treatment time, morbidity, and cost.

Although there is a fear to subject short implants to immediate loading, in the present study, immediate loading proved reliable, with 100% of implant success and survival with a mean of 13.45 months of follow-up. A prospective study [35] reported that four of five short implants (7 mm) placed with immediate loading were lost. However, the literature has limited information about the influence of immediate loading on the survival of short and extra-short implants. Recently, Thomé et al. (2024) [36] reported a 12-month follow-up of two clinical cases with short/extra-short implants in an atrophic posterior mandible, rehabilitated by digital workflow three months after surgery. These cases proved to be a successful treatment alternative.

The high implant survival and success rates in this pilot study can be justified by the abutment/implant interface, with a smooth “one piece” transmucosal collar positioned far from the bone crest to optimize the adaptation of the peri-implant biological space. The smooth surface of the gingival portion allows for more favorable results and minimizes loss of the peri-implant bone crest compared to other prosthetic interface designs [1,15,37]. Since this prosthesis implant interface is at the gingival level, it reduces the presence of bacteria near the implant–bone contact.

A factor that strongly contributed to the implant success rate was the inclusion of patients in a peri-implant follow-up program [38]. Findings suggest that a history of periodontal disease may be a possible risk factor for peri-implant diseases [39]. In this clinical study, 63.64% of patients who received the implants had a history of periodontal disease. However, regularity in follow-up visits was 100%. Lombardo et al. (2020) [21] compared patients with and without a history of periodontal disease in which 326 short and extra-short implants were posterior bimaxillary placed and restored with single-unit prostheses in a 3-year follow-up and concluded that periodontal disease did not appear to negatively influence the peri-implant conditions. When patients’ satisfaction was evaluated, only 36.36% of the patients reported low satisfaction regarding cleaning the prostheses (36.36%), especially when implant-supported. For this reason, it becomes even more essential to guarantee theparticipants maintenance in peri-implant follow-up programs.

When evaluating implant survival, it is necessary to consider peri-implant conditions as stability at the level of the marginal bone crest, since implants with a progressive loss of the bone crest may fail [40]. Previous studies [4,9,22] stated that the highest occlusal load is concentrated in the cervical region of the implant. In this way, bone stress is present around the implant collar and the first few millimeters (5 mm), regardless of the length of the implant. According to results obtained in this pilot study, short implants showed a tendency towards more apical marginal bone loss in the distal and mesial measurement compared to extra-short implants. Studies have shown similar values for marginal bone loss and high survival rates in conventional (8 mm), short (6 mm), and extra-short (4 mm) implants after a 2-year follow-up period [34] and after three years of functional load [1].

The mean mesial C/I ratio was 2.11 and 2.16 in distal. Tang et al. [41] reported a mean clinical C/I ratio of 1.16. The difference from our study can be related to implant size since Tang′s study analyzed only implants with lengths of 8 and 6.5 mm, and ours considered extra-short implants, which can enhance the C/I ratio. The correlation between the C/I ratio and marginal bone loss is controversial. Different studies found no correlation between the C/I ratio and bone loss [42,43,44]; Hingsammer et al. [45] reported that a C/I ratio higher than 1.16 increases the risk of bone loss; and Tang et al. [41] found an inverse correlation between the C/I ratio and bone loss. Malchiodi et al. [46] reported that excessive bone loss can occur only when the clinical C/I ratio exceeds 3.4. The clinical C/I ratio found in our research is less than this threshold. In this way, our study showed a high C/I ratio, 100% implant survival, and marginal bone loss according to the expected, suggesting that short and extra-short implants are an alternative to rehabilitating posterior regions with atrophic alveoli despite their high C/I ratio.

Regarding the soft tissue conditions, the probing depth found in this study varied between 1.76 mm to 2.43 mm for short implants and 1.29 mm to 2.71 mm for extra-short implants. These results are similar to the 2.31 mm reported by Han et al. [47] in one year of post-loading follow-up. Another study showed a 23.9% bleeding on probing in non-diabetic patients, comparable to the bleeding on probing in most of the regions analyzed in this study [48]. In general, our study showed healthy soft tissue around short and extra-short implants.

All mechanical complications found were related to the patients with bruxism. Besides the orientation for occlusal splint use, the patients did not treat the parafunction. It is well-known that overload associated with bruxism can lead to dental wear, screw/implant loosening, screw/implant fracture, and loss of retention [49]. In addition, according to the literature, short implants are associated with higher rates of prosthetic complications compared to conventional implants [50]. As the mechanical complications reported in this study occurred in patients with bruxism, it can be concluded that they are unrelated to the new implant system.

Patients with systemic conditions and smoking habits were not excluded in this study. These characteristics can be confounding in the peri-implant tissue evaluation. The literature has already correlated a smoking habit with higher scores of bleeding index, mean peri-implant probing depth, and peri-implant mucosal inflammation [51]. However, our results showed a good peri-implant tissue, and we believe these confounding did not influence the results due to patient adherence to the maintenance program.

Although this study presents limitations related to sample size and inequality between the number of short and extra-short implants evaluated, we can conclude that these implants showed excellent performance and are an alternative to the rehabilitation of atrophic jaws. Future randomized clinical studies are needed to confirm these results.

## Figures and Tables

**Table 1:** Patient characteristics.

Variable		N	%
Gender	Female	8	72.73
	Male	3	27.27
Systemic condition	No	3	27.27
	Yes	8	72.73
Medication	No	2	18.18
	Yes	9	81.82
Smoking	No	8	72.73
	Yes	3	27.27
Parafunction signs	No	7	63.64
	Yes	4	36.36
History of periodontal disease	Absent	4	36.36
	Present	7	63.64
Bone graft	No	11	100
Implant load	Immediate loading	9	81.82
	Early loading	2	18.18
Implant type	Short	37	84.09
	Extra-short	7	15.91
Bone type	I	8	18.18
	II	20	45.45
	III	16	36.36
Implant insertion torque	30	3	6.82
	32	2	4.55
	45	4	9.09
	60	35	79.55

N = absolute frequency; % = relative frequency; Inf= lower confidence interval; Sup = upper confidence interval.

**Table 2:** Description of prosthesis characteristics and mechanical complications.

				IC 95%	
Variable		N	%	Inf	Sup
Number of tooth changes	0	10	90.91	62.26	98.38
	1	1	9.09	1.62	37.74
Prosthesis material	Acrilic	3	27.27	9.75	56.56
	Metal-ceramic	1	9.09	1.62	37.74
	Hybrid	7	63.64	35.38	84.83
Prosthesis design	Single-unit	2	18.18	5.14	47.7
	Screwed	9	81.82	52.3	94.86
Antagonist	Teeth	1	9.09	1.62	37.74
	Total implant-supported prosthesis	10	90.91	62.26	98.38

N = absolute frequency; % = relative frequency; Inf = lower confidence interval; Sup = upper confidence interval.

**Table 3:** Characteristics of the crown-to-implant ratio C/I < 2 and C/I ≥ 2.

Variable	N (%)	M ± SD
Mesial clinical crown-to-implant ratio		
C/I < 2	18 (40.91)	-
C/I ≥ 2	26 (59.09)	-
Distal clinical crown-to-implant ratio		
C/I < 2	16 (36.36)	-
C/I ≥ 2	28 (63.64)	-
Mesial clinical crown-to-implant ratio		2.11 ± 0.72
Distal clinical crown-to-implant ratio		2.16 ± 0.57

N (%) = absolute and relative frequencies; SD = standard deviation; M = mean.

**Table 4:** Mesial and distal marginal bone loss in short and extra-short implants.

	Implant Type							
	Short				Extra-Short			
Variable	M	MD	SD	IIQ	M	MD	SD	IIQ
Mesial measurement	0.73	0.71	1.14	1.89	−0.1	−0.5	1.16	1.08
Distal measurement	0.86	0.68	1.01	1.78	0.2	0.34	0.71	0.59

M = mean; MD = median; SD = standard deviation; IIQ = interquartile range.

**Table 5:** Distribution of gingival recession and phenotype in each implant type.

		Implant Type			
		Short		Extra-Short	
Variable		N	%	N	%
Gingival recession	Absent	26	70.2	6	85.7
	Present	11	29.8	1	14.3
Gingival phenotype	Thin	18	48.6	2	28.6
	Thick	19	51.4	5	71.4

**Table 6:** Descriptive statistics of probing depth.

	Implant Type							
	Short				Extra-Short			
Variable	M	MD	SD	IIQ	M	MD	SD	IIQ
MB probing depth	2.43	2.00	0.99	1.00	2.71	3.00	0.95	0.50
B probing depth	2.11	2.00	0.99	2.00	1.86	2.00	0.69	0.50
DB probing depth	2.19	2.00	1.17	2.00	1.57	1.00	0.79	1.00
ML probing depth	2.03	2.00	1.32	2.00	1.57	1.00	0.79	1.00
L probing depth	1.81	2.00	1.33	1.00	1.14	1.00	0.38	0.00
DL probing depth	1.76	2.00	1.23	2.00	1.29	1.00	0.49	0.50

N = number of implants; M = mean; MD = median; Min = minimum; Max = maximum; SD = standard deviation; IIQ = interquartile range.

## Data Availability

The original contributions presented in the study are included in the article; further inquiries can be directed to the corresponding author/s.

## References

[1] Estévez-Pérez D., Bustamante-Hernández N., Labaig-Rueda C., Solá-Ruíz M.F., Amengual-Lorenzo J., García-Sala Bonmatí F., Zubizarreta-Macho Á., Agustín-Panadero R. (2020). Comparative Analysis of Peri-Implant Bone Loss in Extra-Short, Short, and Conventional Implants. A 3-Year Retrospective Study. Int. J. Environ. Res. Public. Health.

[2] Sivolella S., Stellini E., Testori T., Di Fiore A., Berengo M., Lops D. (2013). Splinted and Unsplinted Short Implants in Mandibles: A Retrospective Evaluation with 5 to 16 Years of Follow-up. J. Periodontol..

[3] Lombardo G., Marincola M., Signoriello A., Corrocher G., Nocini P.F. (2020). Single-Crown, Short and Ultra-Short Implants, in Association with Simultaneous Internal Sinus Lift in the Atrophic Posterior Maxilla: A Three-Year Retrospective Study. Materials.

[4] Anitua E., Alkhraist M.H., Piñas L., Begoña L., Orive G. (2014). Implant Survival and Crestal Bone Loss around Extra-Short Implants Supporting a Fixed Denture: The Effect of Crown Height Space, Crown-to-Implant Ratio, and Offset Placement of the Prosthesis. Int. J. Oral. Maxillofac. Implant..

[5] Lee S.-A., Lee C.-T., Fu M.M., Elmisalati W., Chuang S.-K. (2014). Systematic Review and Meta-Analysis of Randomized Controlled Trials for the Management of Limited Vertical Height in the Posterior Region: Short Implants (5 to 8 Mm) vs. Longer Implants (>8 Mm) in Vertically Augmented Sites. Int. J. Oral. Maxillofac. Implant..

[6] Adánez M.H., Brezavšček M., Vach K., Fonseca M., Att W. (2018). Clinical and Radiographic Evaluation of Short Implants Placed in the Posterior Mandible: A 1-Year Pilot Split-Mouth Study. J. Oral. Implantol..

[7] Esposito M., Pellegrino G., Pistilli R., Felice P. (2011). Rehabilitation of Postrior Atrophic Edentulous Jaws: Prostheses Supported by 5 Mm Short Implants or by Longer Implants in Augmented Bone? One-Year Results from a Pilot Randomised Clinical Trial. Eur. J. Oral. Implantol..

[8] Calvo-Guirado J.L., Morales-Meléndez H., Pérez-Albacete Martínez C., Morales-Schwarz D., Kolerman R., Fernández-Domínguez M., Gehrke S.A., Maté-Sánchez de Val J.E. (2018). Evaluation of the Surrounding Ring of Two Different Extra-Short Implant Designs in Crestal Bone Maintanence: A Histologic Study in Dogs. Materials.

[9] Hasanoglu Erbasar G.N., Hocaoğlu T.P., Erbasar R.C. (2019). Risk Factors Associated with Short Dental Implant Success: A Long-Term Retrospective Evaluation of Patients Followed up for up to 9 Years. Braz. Oral. Res..

[10] Papaspyridakos P., De Souza A., Vazouras K., Gholami H., Pagni S., Weber H. (2018). Survival Rates of Short Dental Implants (≤6 Mm) Compared with Implants Longer than 6 Mm in Posterior Jaw Areas: A Meta-analysis. Clin. Oral. Implants Res..

[11] Akram Z., Vohra F., Sheikh S.A., Albaijan R., Bukhari I.A., Hussain M. (2019). Clinical and Radiographic Peri-implant Outcomes of Short Dental Implants Placed in Posterior Jaws of Patients with Treated Generalized Aggressive Periodontitis: A 3-year Follow-up Study. Clin. Implant. Dent. Relat. Res..

[12] Telleman G., Raghoebar G.M., Vissink A., Meijer H.J.A. (2014). Impact of Platform Switching on Peri-implant Bone Remodeling around Short Implants in the Posterior Region, 1-year Results from a Split-mouth Clinical Trial. Clin. Implant. Dent. Relat. Res..

[13] Rameh S., Menhall A., Younes R. (2020). Key Factors Influencing Short Implant Success. Oral. Maxillofac. Surg..

[14] Lai H., Si M., Zhuang L., Shen H., Liu Y., Wismeijer D. (2013). Long-term Outcomes of Short Dental Implants Supporting Single Crowns in Posterior Region: A Clinical Retrospective Study of 5–10 Years. Clin. Oral. Implant. Res..

[15] Hernandez-Marcos G., Hernandez-Herrera M., Anitua E. (2018). Marginal Bone Loss Around Short Dental Implants Restored at Implant Level and with Transmucosal Abutment: A Retrospective Study. Int. J. Oral. Maxillofac. Implant..

[16] Misch C.E., Steigenga J., Barboza E., Misch-Dietsh F., Cianciola L.J., Kazor C. (2006). Short Dental Implants in Posterior Partial Edentulism: A Multicenter Retrospective 6-year Case Series Study. J. Periodontol..

[17] Anitua E., Murias-Freijo A., Alkhraisat M.H., Orive G. (2015). Implant-Guided Vertical Bone Augmentation around Extra-Short Implants for the Management of Severe Bone Atrophy. J. Oral. Implantol..

[18] Di Fiore A., Vigolo P., Sivolella S., Cavallin F., Katsoulis J., Monaco C., Stellini E. (2019). Influence of Crown-to-Implant Ratio on Long-Term Marginal Bone Loss Around Short Implants. Int. J. Oral. Maxillofac. Implant..

[19] Malheiros Badaró M., Mendoza Marin D.O., Pauletto P., Simek Vega Gonçalves T.M., Luís Porporatti A., De Luca Canto G. (2021). Failures in Single Extra-Short Implants (≤6 Mm): A Systematic Review and Meta-Analysis. Int. J. Oral Maxillofac. Implant..

[20] Felice P., Barausse C., Pistilli R., Ippolito D.R., Esposito M. (2019). Five-Year Results from a Randomised Controlled Trial Comparing Prostheses Supported by 5-Mm Long Implants or by Longer Implants in Augmented Bone in Posterior Atrophic Edentulous Jaws. Int. J. Oral. Implantol..

[21] Lombardo G., Signoriello A., Marincola M., Nocini P.F. (2020). Assessment of Peri-Implant Soft Tissues Conditions around Short and Ultra-Short Implant-Supported Single Crowns: A 3-Year Retrospective Study on Periodontally Healthy Patients and Patients with a History of Periodontal Disease. Int. J. Environ. Res. Public. Health.

[22] Slotte C., Grønningsaeter A., Halmøy A., Öhrnell L., Stroh G., Isaksson S., Johansson L., Mordenfeld A., Eklund J., Embring J. (2012). Four-millimeter Implants Supporting Fixed Partial Dental Prostheses in the Severely Resorbed Posterior Mandible: Two-year Results. Clin. Implant. Dent. Relat. Res..

[23] Mühlemann H.R., Son S. (1971). Gingival Sulcus Bleeding--a Leading Symptom in Initial Gingivitis. Helv. Odontol. Acta.

[24] Papaspyridakos P., Chen C.-J., Singh M., Weber H.-P., Gallucci G. (2012). Success Criteria in Implant Dentistry: A Systematic Review. J. Dent. Res..

[25] Albrektsson T., Zarb G., Worthington P., Eriksson A.R. (1986). The Long-Term Efficacy of Currently Used Dental Implants: A Review and Proposed Criteria of Success. Int. J Oral Maxillofac Implant..

[26] Lekholm U., Zarb G.A., Branemark P.I., Zarb G.A., Albrektsson T. (1985). Patient Selection and Preparation. Tissue Integrated Prostheses.

[27] Vieira R.A., Melo A.C.M., Budel L.A., Gama J.C., de Mattias Sartori I.A., Thomé G. (2014). Benefits of Rehabilitation with Implants in Masticatory Function: Is Patient Perception of Change in Accordance with the Real Improvement?. J. Oral. Implantol..

[28] R Core Team (2021). A Language and Environment for Statistical Computing 2021.

[29] Pistilli R., Felice P., Cannizzaro G., Piattelli M., Corvino V., Barausse C., Buti J., Soardi E., Esposito M. (2013). Posterior Atrophic Jaws Rehabilitated with Prostheses Supported by 6 Mm Long 4 Mm Wide Implants or by Longer Implants in Augmented Bone. One-Year Post-Loading Results from a Pilot Randomised Controlled Trial. Eur. J. Oral. Implantol..

[30] Esposito M., Pistilli R., Barausse C., Felice P. (2014). Three-Year Results from a Randomised Controlled Trial Comparing Prostheses Supported by 5-Mm Long Implants or by Longer Implants in Augmented Bone in Posterior Atrophic Edentulous Jaws. Eur. J. Oral. Implantol..

[31] Ravidà A., Barootchi S., Askar H., Del Amo F.S.-L., Tavelli L., Wang H.-L. (2019). Long-Term Effectiveness of Extra-Short (≤6 Mm) Dental Implants: A Systematic Review. Int. J. Oral. Maxillofac. Implant..

[32] Shah S.N., Chung J., Kim D.M., Machtei E.E. (2018). Can Extra-Short Dental Implants Serve as Alternatives to Bone Augmentation? A Preliminary Longitudinal Randomized Controlled Clinical Trial. Quintessence Int..

[33] Al-Johany S.S. (2019). Survival Rates of Short Dental Implants (≤6.5 Mm) Placed in Posterior Edentulous Ridges and Factors Affecting Their Survival After a 12-Month Follow-up Period: Systematic Review. Int. J. Oral. Maxillofac. Implant..

[34] Torassa D., Naldini P., Calvo-Guirado J.L., Fernández-Bodereau E. (2020). Prospective, Clinical Pilot Study with Eleven 4-Mm Extra-Short Implants Splinted to Longer Implants for Posterior Maxilla Rehabilitation. J. Clin. Med..

[35] Grunder U., Polizzi G., Goené R., Hatano N., Henry P., Jackson W.J., Kawamura K., Köhler S., Renouard F., Rosenberg R. (1999). A 3-Year Prospective Multicenter Follow-up Report on the Immediate and Delayed-Immediate Placement of Implants. Int. J. Oral. Maxillofac. Implant..

[36] Thomé G., Bernardes S.R., Cartelli C., Uhlendorf J., Deliberador T.M. (2024). Digital Workflow for New Extra-Short and Short Implants in Atrophic Posterior Mandible Rehabilitation. A Case Report with One-Year of Follow-Up. J. Clin. Med. Images.

[37] Tesmer M., Wallet S., Koutouzis T., Lundgren T. (2009). Bacterial Colonization of the Dental Implant Fixture–Abutment Interface: An in Vitro Study. J. Periodontol..

[38] Berglundh T., Armitage G., Araujo M.G., Avila-Ortiz G., Blanco J., Camargo P.M., Chen S., Cochran D., Derks J., Figuero E. (2018). Peri-implant Diseases and Conditions: Consensus Report of Workgroup 4 of the 2017 World Workshop on the Classification of Periodontal and Peri-Implant Diseases and Conditions. J. Periodontol..

[39] Klokkevold P.R., Han T.J. (2007). How Do Smoking, Diabetes, and Periodontitis Affect Outcomes of Implant Treatment?. Int. J. Oral Maxillofac. Implant..

[40] Coli P., Christiaens V., Sennerby L., De Bruyn H. (2017). Reliability of Periodontal Diagnostic Tools for Monitoring Peri-implant Health and Disease. Periodontol. 2000.

[41] Tang Y., Yu H., Wang J., Gao M., Qiu L. (2020). Influence of Crown-to-implant Ratio and Different Prosthetic Designs on the Clinical Conditions of Short Implants in Posterior Regions: A 4-year Retrospective Clinical and Radiographic Study. Clin. Implant. Dent. Relat. Res..

[42] Hof M., Pommer B., Zukic N., Vasak C., Lorenzoni M., Zechner W. (2015). Influence of Prosthetic Parameters on Peri-implant Bone Resorption in the First Year of Loading: A Multi-factorial Analysis. Clin. Implant. Dent. Relat. Res..

[43] Meijer H.J.A., Boven C., Delli K., Raghoebar G.M. (2018). Is There an Effect of Crown-to-implant Ratio on Implant Treatment Outcomes? A Systematic Review. Clin. Oral. Implant. Res..

[44] Garaicoa-Pazmiño C., Suárez-López del Amo F., Monje A., Catena A., Ortega-Oller I., Galindo-Moreno P., Wang H. (2014). Influence of Crown/Implant Ratio on Marginal Bone Loss: A Systematic Review. J. Periodontol..

[45] Hingsammer L., Watzek G., Pommer B. (2017). The Influence of Crown-to-implant Ratio on Marginal Bone Levels around Splinted Short Dental Implants: A Radiological and Clincial Short Term Analysis. Clin. Implant. Dent. Relat. Res..

[46] Malchiodi L., Cucchi A., Ghensi P., Consonni D., Nocini P.F. (2014). Influence of Crown–Implant Ratio on Implant Success Rates and Crestal Bone Levels: A 36-month Follow-up Prospective Study. Clin. Oral. Implant. Res..

[47] Han J., Tang Z., Zhang X., Meng H. (2018). A Prospective, Multi-center Study Assessing Early Loading with Short Implants in Posterior Regions. A 3-year Post-loading Follow-up Study. Clin. Implant. Dent. Relat. Res..

[48] Mokeem S., Alfadda S.A., Al-Shibani N., Alrabiah M., Al-Hamdan R.S., Vohra F., Abduljabbar T. (2019). Clinical and Radiographic Peri-implant Variables around Short Dental Implants in Type 2 Diabetic, Prediabetic, and Non-diabetic Patients. Clin. Implant. Dent. Relat. Res..

[49] Zhou Y., Gao J., Luo L., Wang Y. (2016). Does Bruxism Contribute to Dental Implant Failure? A Systematic Review and Meta-analysis. Clin. Implant. Dent. Relat. Res..

[50] Cruz R.S., Lemos C.A.d.A., Batista V.E.d.S., Gomes J.M.d.L., Pellizzer E.P., Verri F.R. (2018). Short Implants versus Longer Implants with Maxillary Sinus Lift. A Systematic Review and Meta-Analysis. Braz. Oral. Res..

[51] Haas R., Haimböck W., Mailath G., Watzek G. (1996). The Relationship of Smoking on Peri-Implant Tissue: A Retrospective Study. J. Prosthet. Dent..

